# The COVID-19 Pandemic: Reshaping Public Health Policy Response Envisioning Health as a Common Good

**DOI:** 10.3390/ijerph19169985

**Published:** 2022-08-12

**Authors:** Domenico Vito, Paolo Lauriola, Clelia D’Apice

**Affiliations:** 1 Metabolism of Cities Living Lab, San Diego State University, San Diego, CA 92182, USA; 2 Italian Network of Sentinel Phycians for the Environment (RIMSA), International Society Doctors for the Environment (ISDE), Federazione Nazione Ordine dei Medici (FNOMCeO), 52100 Arezzo, Italy; 3 Department of Medicine and Surgery, University of Parma, 43125 Parma, Italy

On 31 December 2019, the World Health Organization (WHO) was informed of a cluster of cases of pneumonia of unknown cause detected in Wuhan, Hubei Province, China. On 12 January 2020, it was announced that a novel coronavirus had been identified in samples obtained from cases, and that an initial analysis of virus genetic sequences suggested that this was the cause of the outbreak. This virus has been referred to as SARS-CoV-2, and the associated disease as COVID-19.

In most countries, public health response to the COVID-19 outbreak has required the efforts of a large proportion of the public health workforce, alongside most other sectors of society. The environmental aspects of the COVID-19 origin, spread, and pattern of clinical severity have not been completely clarified. Improved understanding of these aspects could play a role both as part of the response and of preparedness, and as a component of the efforts to advance the scientific basis of public health role and practice.

In 2004, the Wildlife Conservation Society in the Manhattan Principles defined the ‘One World One Health’ paradigm. [1] Such a statement has been confirmed and strengthened by the Tripartite and UNEP support OHHLEP’s definition of “One Health”. [2] The One Health approach to global health security recommends a holistic view of the interface between human, animal, and ecosystem health domains. One clear advantage of implementing a One Health strategy is highlighted by the cost-effectiveness of early detection in animals, thus reducing the impact on human lives (Figure 1) and adding genuine value.

Since the COVID-19 pandemic has been quickly linked to the relationships between humankind, animals, and the environment, such an approach immediately became the overarching issue that drove any interpretations and actions to address such a dramatic global event.

According to such a relationship, which has been now finally confirmed, [4] this Special Issue has been conceived.

The COVID-19 Pandemic: Reshaping Public Health Policy Response Envisioning Health as a Common Good.

According to Benach (2021) [5], “COVID-19 not only constitutes a serious public health problem and a global major threat to the poorest and most vulnerable social groups and neighborhoods of the world, creating a potential pandemic of inequality, but also poses an enormous challenge from the perspective of public health, ethics, economy, environment, and politics. However, many of the deep and complex systemic interrelationships created and developed by this pandemic are largely hidden, unknown, or neglected, both by the hegemonic media and by a highly specialized and fragmented academic world. However, when all the available knowledge will be critically integrated, the origins and effects underlying this pandemic are likely to be found in the development of neoliberal capitalism and its inherent logic of ceaseless accumulation, economic growth, large inequalities, and ecological devastation”.

The COVID-19 pandemic is triggering two parallel crises. The first is clearly a health crisis, with hundreds of thousands of deaths from the direct effects of the disease, showing how health systems throughout the world were dramatically unprepared in tackling such a health emergency. The second is an economic crisis, with important effects on the markets throughout the world, showing how health and economics are intrinsically linked, thus requiring coordinated policies. Further, the effects of the COVID-19 pandemic extend to many aspects such as inequalities, environment, ethic, thus triggering an environmental and social crisis. However, the COVID-19-related crises provided clear evidence on how a biotic element can disrupt human related activities in an extensive manner. For such reasons, the COVID-19 health crisis showed several similarities with the climate crisis, and both foster the activation of societies’ community resilience that led to the need for radical changes [6].

The COVID-19 pandemic has required governments and policymakers to challenge their limits in terms of preparedness and response, facing their biggest test in generations. Still, the pandemic represents an opportunity for governments and policymakers—but also for economics, universities, health systems and the private sector—to tackle underlying problems besetting health systems and ameliorate themselves through more inclusive, fair, and coordinated public health policies and best practices.

By spreading all over the world and representing a common challenge, the COVID-19 pandemic has, in some ways, united the human race, embracing it in a sole huge tragedy. However, it has also highlighted and accentuated incredible inequalities and inequities.

The COVID-19 pandemic has shown that the solution to the pandemic cannot be achieved independently nor by individual states, but it must necessarily involve the entire planet. Thus, the COVID-19 crisis urgently calls us to see health as a common and global good, hence requiring a broader perspective and more coordinated efforts among governments and policymakers across the world [7].

Socio-economic drivers must also be taken into proper account. To be more resilient to the not-at-all-improbable upcoming crisis, academics, entrepreneurs, and policy makers should promote theoretical efforts aiming at providing the means to global economies to adopt “multiple forms of value and political work that embeds these theories in societal institutions” [8]. Such a need must also consider the urgency of the impending climate crisis, as “Nature is sending us a message” [9] by means of COVID-19.

According to The Lancet (2021) “In 2020, a virus that thrived on chronic disease and inequality became the great ‘revealer’. COVID-19 revealed the fragility of civilizations built on social injustices, short-term policies, and a dangerous disregard for the environment. The need to become more resilient to crises of all kinds is almost universally agreed on. But to construct that resilience, a philosophical change in how we care for each other, and our environment must be made. COVID-19 has proven that the economic and political success of individual countries is founded on the health of its population” [10].

Simply put, the COVID-19 pandemic clearly unveiled health as the fundamental driver of the economic, cultural, and social development of our society, as it is the first and foremost “*common good*” either at the individual or community level [11]. 

This Special Issue called for contributions aimed at providing ideas and proposals to reshape public health policies and health systems in a more equal, resilient, and coordinated way. In particular, the Special Issue invited researchers to reflect on our society in view of a new and more equitable and sustainable future.

Authors responded to the call with several proposals. Dasgupta et al. underlined one of the indirect but nonetheless essential side effects of the COVID-19 pandemic, food insecurity (FI) in nine sub-Saharan countries by taking a multi-country cross-sectional picture. They investigated the socioeconomic determinants of FI through an econometric model. Such analysis revealed that female-headed households, the poor, and the less formally educated, appear to suffer more in terms of food insecurity during this global pandemic, and there is considerable spatial heterogeneity within country food insecurity, suggesting that tailored policies will be required. Finally, their findings can also be used as lessons to reshape policies to tackle the heterogeneous impacts of climate change.

One crucial aspect that affects the difficulties to face health threats in Africa is the lack of an effective pharmaceutical system across the whole continent. Ussai et al. investigated Africa’s vulnerability during the COVID-19 pandemic due to its reliance on imports for most vaccines, medicines, and other health product needs. They summarized the lesson learnt from COVID-19: (i) the urgency of strengthening regional and national regulatory systems, (ii) a clear demand for supporting the local manufacturing in the context of the Manufacturing Plan for Africa and the operationalization of the African Medicines Agency, and (iii) the equal importance of advancing vaccine product-related value chain mapping at country level.

Public policies could be the defining factor in shaping people’s wellbeing and quality of life, especially amid global health crises such as COVID-19. As such, Su conducted a literature review on a data-driven and digitally enabled policy-making approach. The review focused on three topics: (1) the advantages of public policies based on big, smart, and up-to-date data, and (2) the effects of data-driven and digitally enabled policy making in terms of its anticipated outcomes and unintended consequences. (3) In such a way, he provided detailed information on how this method could help policymakers to effectively establish laws and regulations based on virtual public participation amid and beyond COVID-19, and in turn, avoid or mitigate potential unintended consequences (divorces, mental distress, anxiety, depression, to suicide behaviors). The smart exploitation of data has been another crucial point in the management of the pandemic: the availability of a large amount of multivariate data has contributed to the development of data-oriented strategies and responsive adaptive mechanisms based on data analysis and mining, as proposed by Ying et.al and Fernandez et al.

The COVID-19 pandemic has shed light on the critical nexus between public policy and public health and the implications that policy change has for public health and health inequities. Jackson et al. introduce the creation and potential areas of application of a California- and US-based COVID-19 policy database. The granular detail in this database can form the basis for analyses that leverage variation across cities, counties, and time. This evidence will enable future policy evaluations to quantify the impact of policy changes on health disparities and inform potential interventions to address inequities.

Ghazali et al. described the characteristics of COVID-19 cases and close contacts during the first wave of COVID-19 in Malaysia (23 January 2020 to 26 February 2020) and analyzed the reasons why the outbreak did not continue to spread and lessons that can be learnt from this experience. They applied an extended susceptible–exposed–infectious–removed (SEIR) model using COVID-19 case data to determine the basic reproduction number and trajectory of cases during the first wave. Owing to the early case detection, active screening, extensive contact tracing, testing and prompt isolation/quarantine, the outbreak was contained and controlled during the first wave.

In conclusion, the COVID-19 health crisis is suggestive of a new generation of diseases. It reveals the several inter-connections between human and planetary health. The experience of COVID-19 taught us that combining environment and human health is more urgent than ever. In other words, in the case of COVID-19, changes such as the climatic ones, even if triggered by regional or local political choices such as deforestation, intensive farming, and energy production based on an “all-and-now” profit approach, have rapidly affected all humankind.

We hope this collection sheds light on health as a common good, inducing policy makers and citizens all over the world to reflect on our planet’s future.

## Figures and Tables

**Figure 1:**
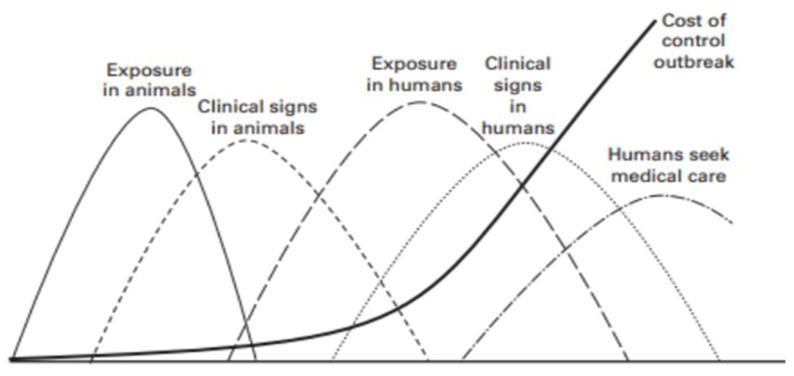
Relationship between time of detection of emerging zoonotic disease and total cost of outbreak. Source: The World Bank, 2012 [3].
